# Correction to Reapplication of the Type IV Hypersensitivity Quantitative Risk Assessment to Assess Ingredients Used on Canines

**DOI:** 10.1002/vms3.70572

**Published:** 2025-08-18

McDermott, A., G. Lorch, J. Nash, and P. Kern. 2025. “Reapplication of the Type IV Hypersensitivity Quantitative Risk Assessment to Assess Ingredients Used on Canines.” Veterinary Medicine and Science 11: e70463. https://doi.org/10.1002/vms3.70463

Dr. Gwendolen Lorch's affiliation was published incorrectly. Her current affiliation should be: Department of Dermatology, MedVet Columbus, Worthington, Ohio, USA.

We apologize for this error.

